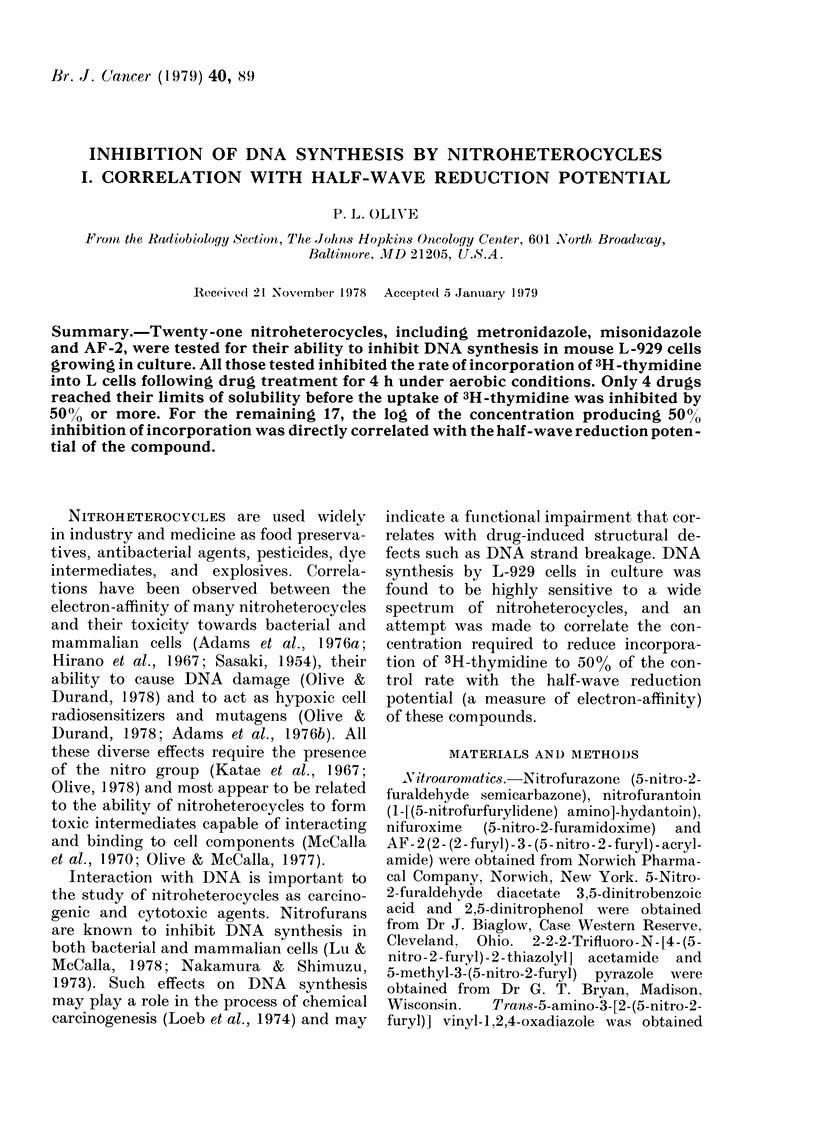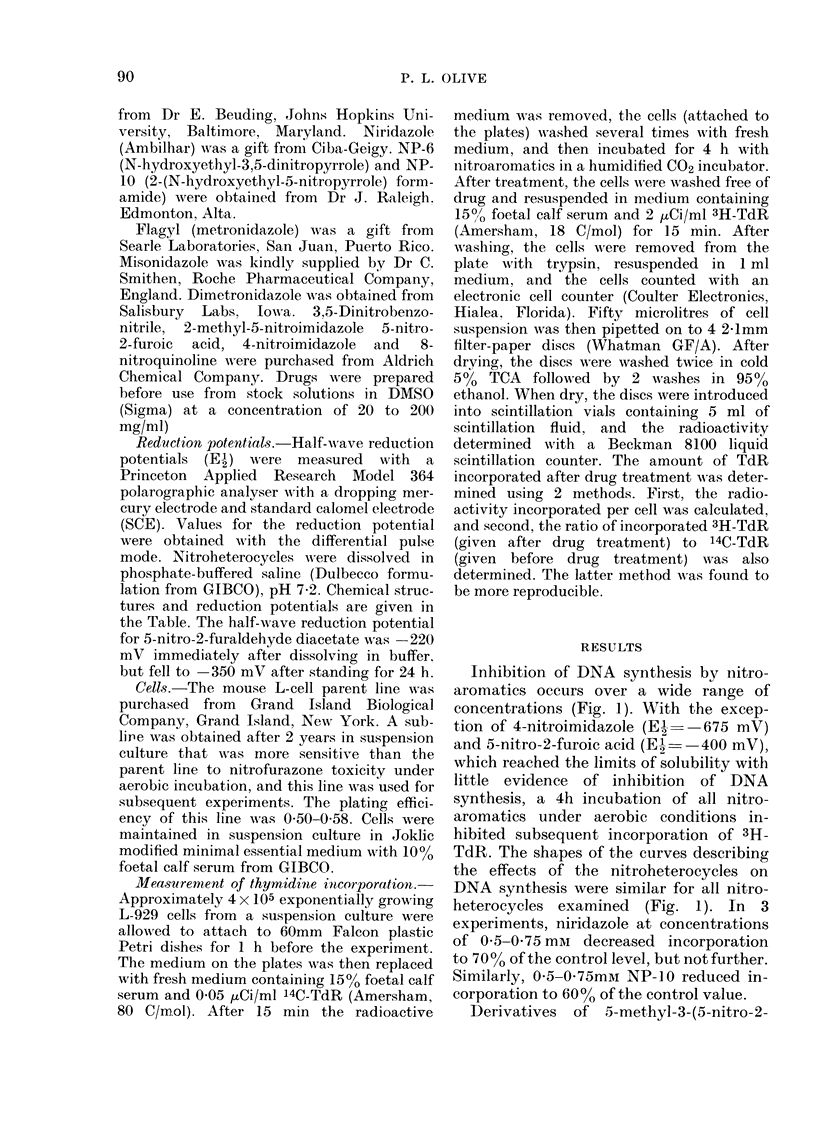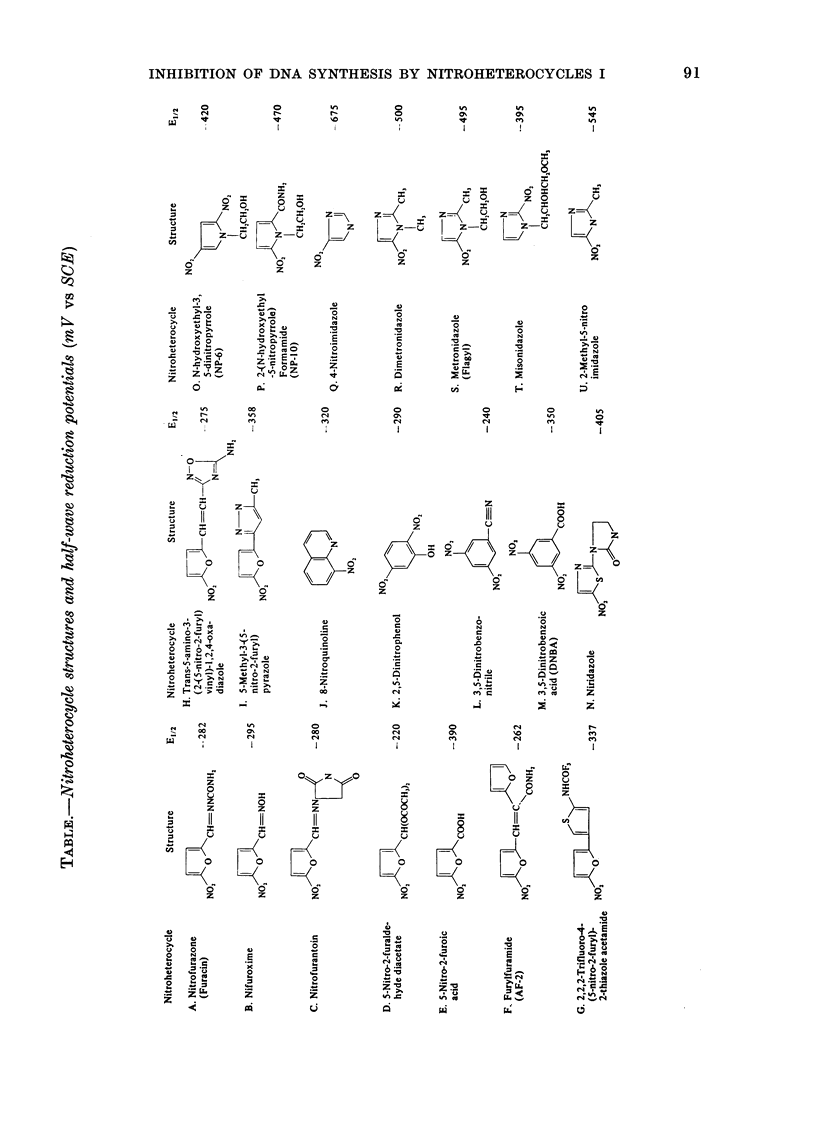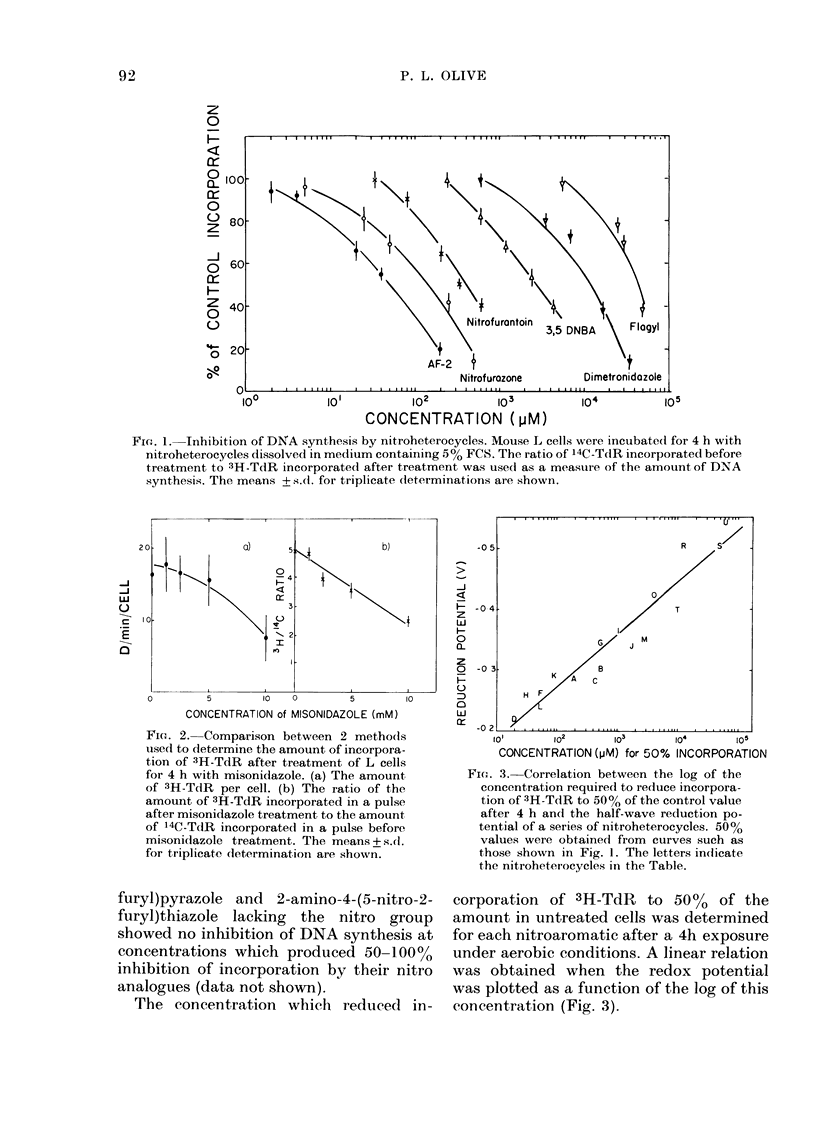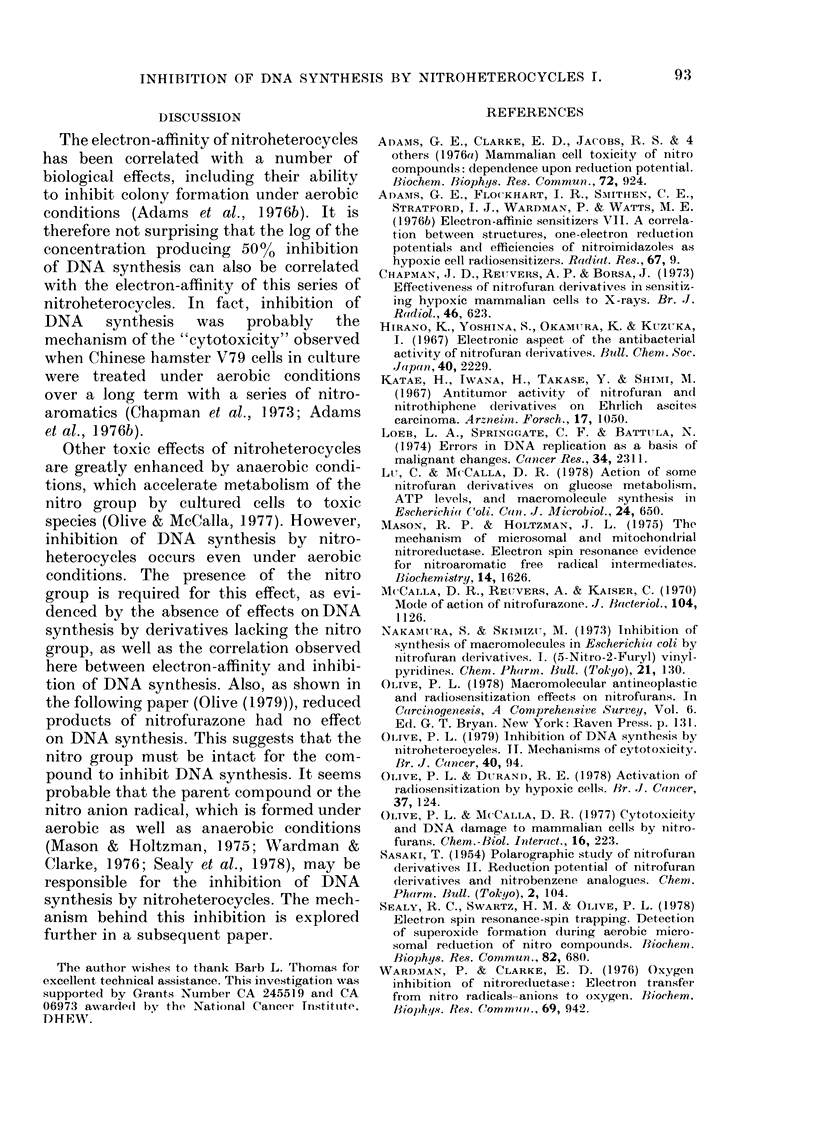# Inhibition of DNA synthesis by nitroheterocycles. I. Correlation with half-wave reduction potential.

**DOI:** 10.1038/bjc.1979.144

**Published:** 1979-07

**Authors:** P. L. Olive

## Abstract

Twenty-one nitroheterocycles, including metronidazole, misonidazole and AF-2, were tested for their ability to inhibit DNA synthesis in mouse L-929 cells growing in culture. All those tested inhibited the rate of incorporation of 3H-thymidine into L cells following drug treatment for 4 h under aerobic conditions. Only 4 drugs reached their limits of solubility before the uptake of 3H-thymidine was inhibited by 50% or more. For the remaining 17, the log of the concentration producing 50% inhibition of incorporation was directly correlated with the half-wave reduction potential of the compound.


Br. J. Cancer (1979) 40, 89

### INHIBITION OF DNA SYNTHESIS BY NITROHETEROCYCLES I. CORRELATION WITH HALF-WAVE REDUCTION POTENTIAL

P. L. OLIVE

*From the Radiobiology Section, The Johns Hopkins Oncology Center, 601 North Broadway, Baltimore, MD 21205, U.S.A.*

Received 21 November 1978 Accepted 5 January 1979

**Summary.—Twenty-one nitroheterocycles, including metronidazole, misonidazole and AF-2, were tested for their ability to inhibit DNA synthesis in mouse L-929 cells growing in culture. All those tested inhibited the rate of incorporation of $^3H$-thymidine into L cells following drug treatment for 4 h under aerobic conditions. Only 4 drugs reached their limits of solubility before the uptake of $^3H$-thymidine was inhibited by 50% or more. For the remaining 17, the log of the concentration producing 50% inhibition of incorporation was directly correlated with the half-wave reduction potential of the compound.**

NITROHETEROCYCLES are used widely in industry and medicine as food preservatives, antibacterial agents, pesticides, dye intermediates, and explosives. Correlations have been observed between the electron-affinity of many nitroheterocycles and their toxicity towards bacterial and mammalian cells (Adams *et al.*, 1976*a*; Hirano *et al.*, 1967; Sasaki, 1954), their ability to cause DNA damage (Olive & Durand, 1978) and to act as hypoxic cell radiosensitizers and mutagens (Olive & Durand, 1978; Adams *et al.*, 1976*b*). All these diverse effects require the presence of the nitro group (Katae *et al.*, 1967; Olive, 1978) and most appear to be related to the ability of nitroheterocycles to form toxic intermediates capable of interacting and binding to cell components (McCalla *et al.*, 1970; Olive & McCalla, 1977).

Interaction with DNA is important to the study of nitroheterocycles as carcinogenic and cytotoxic agents. Nitrofurans are known to inhibit DNA synthesis in both bacterial and mammalian cells (Lu & McCalla, 1978; Nakamura & Shimuzu, 1973). Such effects on DNA synthesis may play a role in the process of chemical carcinogenesis (Loeb *et al.*, 1974) and may indicate a functional impairment that correlates with drug-induced structural defects such as DNA strand breakage. DNA synthesis by L-929 cells in culture was found to be highly sensitive to a wide spectrum of nitroheterocycles, and an attempt was made to correlate the concentration required to reduce incorporation of $^3H$-thymidine to 50% of the control rate with the half-wave reduction potential (a measure of electron-affinity) of these compounds.

### MATERIALS AND METHODS

*Nitroaromatics.*—Nitrofurazone (5-nitro-2-furaldehyde semicarbazone), nitrofurantoin (1-[(5-nitrofurfurylidene) amino]-hydantoin), nifuroxime (5-nitro-2-furamidoxime) and AF-2(2-(2-furyl)-3-(5-nitro-2-furyl)-acrylamide) were obtained from Norwich Pharmacal Company, Norwich, New York. 5-Nitro-2-furaldehyde diacetate 3,5-dinitrobenzoic acid and 2,5-dinitrophenol were obtained from Dr J. Biaglow, Case Western Reserve, Cleveland, Ohio. 2-2-2-Trifluoro-N-[4-(5-nitro-2-furyl)-2-thiazolyl] acetamide and 5-methyl-3-(5-nitro-2-furyl) pyrazole were obtained from Dr G. T. Bryan, Madison, Wisconsin. *Trans*-5-amino-3-[2-(5-nitro-2-furyl)] vinyl-1,2,4-oxadiazole was obtained from Dr E. Beuding, Johns Hopkins University, Baltimore, Maryland. Niridazole (Ambilhar) was a gift from Ciba-Geigy. NP-6 (N-hydroxyethyl-3,5-dinitropyrrole) and NP-10 (2-(N-hydroxyethyl-5-nitropyrrole) formamide) were obtained from Dr J. Raleigh, Edmonton, Alta.

Flagyl (metronidazole) was a gift from Searle Laboratories, San Juan, Puerto Rico. Misonidazole was kindly supplied by Dr C. Smithen, Roche Pharmaceutical Company, England. Dimetronidazole was obtained from Salisbury Labs, Iowa. 3,5-Dinitrobenzonitrile, 2-methyl-5-nitroimidazole 5-nitro-2-furoic acid, 4-nitroimidazole and 8-nitroquinoline were purchased from Aldrich Chemical Company. Drugs were prepared before use from stock solutions in DMSO (Sigma) at a concentration of 20 to 200 mg/ml)

*Reduction potentials.*—Half-wave reduction potentials ($E\frac{1}{2}$) were measured with a Princeton Applied Research Model 364 polarographic analyser with a dropping mercury electrode and standard calomel electrode (SCE). Values for the reduction potential were obtained with the differential pulse mode. Nitroheterocycles were dissolved in phosphate-buffered saline (Dulbecco formulation from GIBCO), pH 7·2. Chemical structures and reduction potentials are given in the Table. The half-wave reduction potential for 5-nitro-2-furaldehyde diacetate was −220 mV immediately after dissolving in buffer, but fell to −350 mV after standing for 24 h.

*Cells.*—The mouse L-cell parent line was purchased from Grand Island Biological Company, Grand Island, New York. A subline was obtained after 2 years in suspension culture that was more sensitive than the parent line to nitrofurazone toxicity under aerobic incubation, and this line was used for subsequent experiments. The plating efficiency of this line was 0·50–0·58. Cells were maintained in suspension culture in Joklic modified minimal essential medium with 10% foetal calf serum from GIBCO.

*Measurement of thymidine incorporation.*—Approximately $4 \times 10^5$ exponentially growing L-929 cells from a suspension culture were allowed to attach to 60mm Falcon plastic Petri dishes for 1 h before the experiment. The medium on the plates was then replaced with fresh medium containing 15% foetal calf serum and 0·05 $\mu$Ci/ml $^{14}$C-TdR (Amersham, 80 C/mol). After 15 min the radioactive medium was removed, the cells (attached to the plates) washed several times with fresh medium, and then incubated for 4 h with nitroaromatics in a humidified $CO_2$ incubator. After treatment, the cells were washed free of drug and resuspended in medium containing 15% foetal calf serum and 2 $\mu$Ci/ml $^3$H-TdR (Amersham, 18 C/mol) for 15 min. After washing, the cells were removed from the plate with trypsin, resuspended in 1 ml medium, and the cells counted with an electronic cell counter (Coulter Electronics, Hialea, Florida). Fifty microlitres of cell suspension was then pipetted on to 4 2·1mm filter-paper discs (Whatman GF/A). After drying, the discs were washed twice in cold 5% TCA followed by 2 washes in 95% ethanol. When dry, the discs were introduced into scintillation vials containing 5 ml of scintillation fluid, and the radioactivity determined with a Beckman 8100 liquid scintillation counter. The amount of TdR incorporated after drug treatment was determined using 2 methods. First, the radioactivity incorporated per cell was calculated, and second, the ratio of incorporated $^3$H-TdR (given after drug treatment) to $^{14}$C-TdR (given before drug treatment) was also determined. The latter method was found to be more reproducible.

### RESULTS

Inhibition of DNA synthesis by nitroaromatics occurs over a wide range of concentrations (Fig. 1). With the exception of 4-nitroimidazole ($E\frac{1}{2} = -675$ mV) and 5-nitro-2-furoic acid ($E\frac{1}{2} = -400$ mV), which reached the limits of solubility with little evidence of inhibition of DNA synthesis, a 4h incubation of all nitroaromatics under aerobic conditions inhibited subsequent incorporation of $^3$H-TdR. The shapes of the curves describing the effects of the nitroheterocycles on DNA synthesis were similar for all nitroheterocycles examined (Fig. 1). In 3 experiments, niridazole at concentrations of 0·5–0·75 mM decreased incorporation to 70% of the control level, but not further. Similarly, 0·5–0·75mM NP-10 reduced incorporation to 60% of the control value.

Derivatives of 5-methyl-3-(5-nitro-2-

TABLE.—*Nitroheterocycle structures and half-wave reduction potentials* (*mV* vs *SCE*)

| Nitroheterocycle | Structure | $E_{1/2}$ | Nitroheterocycle | Structure | $E_{1/2}$ | Nitroheterocycle | Structure | $E_{1/2}$ |
|---|---|---|---|---|---|---|---|---|
| A. Nitrofurazone (Furacin) |  | −282 | H. Trans-5-amino-3-(2-(5-nitro-2-furyl) vinyl)-1,2,4-oxadiazole |  | −275 | O. N-hydroxyethyl-3, 5-dinitropyrrole (NP-6) |  | −420 |
| B. Nifuroxime |  | −295 | I. 5-Methyl-3-(5-nitro-2-furyl) pyrazole |  | −358 | P. 2-(N-hydroxyethyl -5-nitropyrrole) Formamide (NP-10) |  | −470 |
| C. Nitrofurantoin |  | −280 | J. 8-Nitroquinoline |  | −320 | Q. 4-Nitroimidazole |  | −675 |
| D. 5-Nitro-2-furaldehyde diacetate |  | −220 | K. 2,5-Dinitrophenol |  | −290 | R. Dimetronidazole |  | −500 |
| E. 5-Nitro-2-furoic acid |  | −390 | L. 3,5-Dinitrobenzonitrile |  | −240 | S. Metronidazole (Flagyl) |  | −495 |
| F. Furylfuramide (AF-2) |  | −262 | M. 3,5-Dinitrobenzoic acid (DNBA) |  | −350 | T. Misonidazole |  | −395 |
| G. 2,2,2-Trifluoro-4-(5-nitro-2-furyl)-2-thiazole acetamide |  | −337 | N. Niridazole |  | −405 | U. 2-Methyl-5-nitro imidazole |  | −545 |

FIG. 1.—Inhibition of DNA synthesis by nitroheterocycles. Mouse L cells were incubated for 4 h with nitroheterocycles dissolved in medium containing 5% FCS. The ratio of $^{14}$C-TdR incorporated before treatment to $^{3}$H-TdR incorporated after treatment was used as a measure of the amount of DNA synthesis. The means ±s.d. for triplicate determinations are shown.

FIG. 2.—Comparison between 2 methods used to determine the amount of incorporation of $^{3}$H-TdR after treatment of L cells for 4 h with misonidazole. (a) The amount of $^{3}$H-TdR per cell. (b) The ratio of the amount of $^{3}$H-TdR incorporated in a pulse after misonidazole treatment to the amount of $^{14}$C-TdR incorporated in a pulse before misonidazole treatment. The means ± s.d. for triplicate determination are shown.

FIG. 3.—Correlation between the log of the concentration required to reduce incorporation of $^{3}$H-TdR to 50% of the control value after 4 h and the half-wave reduction potential of a series of nitroheterocycles. 50% values were obtained from curves such as those shown in Fig. 1. The letters indicate the nitroheterocycles in the Table.

furyl)pyrazole and 2-amino-4-(5-nitro-2-furyl)thiazole lacking the nitro group showed no inhibition of DNA synthesis at concentrations which produced 50–100% inhibition of incorporation by their nitro analogues (data not shown).

The concentration which reduced incorporation of $^{3}$H-TdR to 50% of the amount in untreated cells was determined for each nitroaromatic after a 4h exposure under aerobic conditions. A linear relation was obtained when the redox potential was plotted as a function of the log of this concentration (Fig. 3).

### DISCUSSION

The electron-affinity of nitroheterocycles has been correlated with a number of biological effects, including their ability to inhibit colony formation under aerobic conditions (Adams *et al.*, 1976*b*). It is therefore not surprising that the log of the concentration producing 50% inhibition of DNA synthesis can also be correlated with the electron-affinity of this series of nitroheterocycles. In fact, inhibition of DNA synthesis was probably the mechanism of the "cytotoxicity" observed when Chinese hamster V79 cells in culture were treated under aerobic conditions over a long term with a series of nitroaromatics (Chapman *et al.*, 1973; Adams *et al.*, 1976*b*).

Other toxic effects of nitroheterocycles are greatly enhanced by anaerobic conditions, which accelerate metabolism of the nitro group by cultured cells to toxic species (Olive & McCalla, 1977). However, inhibition of DNA synthesis by nitroheterocycles occurs even under aerobic conditions. The presence of the nitro group is required for this effect, as evidenced by the absence of effects on DNA synthesis by derivatives lacking the nitro group, as well as the correlation observed here between electron-affinity and inhibition of DNA synthesis. Also, as shown in the following paper (Olive (1979)), reduced products of nitrofurazone had no effect on DNA synthesis. This suggests that the nitro group must be intact for the compound to inhibit DNA synthesis. It seems probable that the parent compound or the nitro anion radical, which is formed under aerobic as well as anaerobic conditions (Mason & Holtzman, 1975; Wardman & Clarke, 1976; Sealy *et al.*, 1978), may be responsible for the inhibition of DNA synthesis by nitroheterocycles. The mechanism behind this inhibition is explored further in a subsequent paper.

The author wishes to thank Barb L. Thomas for excellent technical assistance. This investigation was supported by Grants Number CA 245519 and CA 06973 awarded by the National Cancer Institute, DHEW.

### REFERENCES

Adams, G. E., Clarke, E. D., Jacobs, R. S. & 4 others (1976*a*) Mammalian cell toxicity of nitro compounds: dependence upon reduction potential. *Biochem. Biophys. Res. Commun.*, **72**, 924.

Adams, G. E., Flockhart, I. R., Smithen, C. E., Stratford, I. J., Wardman, P. & Watts, M. E. (1976*b*) Electron-affinic sensitizers VII. A correlation between structures, one-electron reduction potentials and efficiencies of nitroimidazoles as hypoxic cell radiosensitizers. *Radiat. Res.*, **67**, 9.

Chapman, J. D., Reuvers, A. P. & Borsa, J. (1973) Effectiveness of nitrofuran derivatives in sensitizing hypoxic mammalian cells to X-rays. *Br. J. Radiol.*, **46**, 623.

Hirano, K., Yoshina, S., Okamura, K. & Kuzuka, I. (1967) Electronic aspect of the antibacterial activity of nitrofuran derivatives. *Bull. Chem. Soc. Japan*, **40**, 2229.

Katae, H., Iwana, H., Takase, Y. & Shimi, M. (1967) Antitumor activity of nitrofuran and nitrothiphene derivatives on Ehrlich ascites carcinoma. *Arzneim. Forsch.*, **17**, 1050.

Loeb, L. A., Springgate, C. F. & Battula, N. (1974) Errors in DNA replication as a basis of malignant changes. *Cancer Res.*, **34**, 2311.

Lu, C. & McCalla, D. R. (1978) Action of some nitrofuran derivatives on glucose metabolism, ATP levels, and macromolecule synthesis in *Escherichia Coli*. *Can. J. Microbiol.*, **24**, 650.

Mason, R. P. & Holtzman, J. L. (1975) The mechanism of microsomal and mitochondrial nitroreductase. Electron spin resonance evidence for nitroaromatic free radical intermediates. *Biochemistry*, **14**, 1626.

McCalla, D. R., Reuvers, A. & Kaiser, C. (1970) Mode of action of nitrofurazone. *J. Bacteriol.*, **104**, 1126.

Nakamura, S. & Skimizu, M. (1973) Inhibition of synthesis of macromolecules in *Escherichia coli* by nitrofuran derivatives. I. (5-Nitro-2-Furyl) vinylpyridines. *Chem. Pharm. Bull.* (*Tokyo*), **21**, 130.

Olive, P. L. (1978) Macromolecular antineoplastic and radiosensitization effects on nitrofurans. In *Carcinogenesis, A Comprehensive Survey*, Vol. 6. Ed. G. T. Bryan. New York: Raven Press. p. 131.

Olive, P. L. (1979) Inhibition of DNA synthesis by nitroheterocycles. II. Mechanisms of cytotoxicity. *Br. J. Cancer*, **40**, 94.

Olive, P. L. & Durand, R. E. (1978) Activation of radiosensitization by hypoxic cells. *Br. J. Cancer*, **37**, 124.

Olive, P. L. & McCalla, D. R. (1977) Cytotoxicity and DNA damage to mammalian cells by nitrofurans. *Chem.-Biol. Interact.*, **16**, 223.

Sasaki, T. (1954) Polarographic study of nitrofuran derivatives II. Reduction potential of nitrofuran derivatives and nitrobenzene analogues. *Chem. Pharm. Bull.* (*Tokyo*), **2**, 104.

Sealy, R. C., Swartz, H. M. & Olive, P. L. (1978) Electron spin resonance-spin trapping. Detection of superoxide formation during aerobic microsomal reduction of nitro compounds. *Biochem. Biophys. Res. Commun.*, **82**, 680.

Wardman, P. & Clarke, E. D. (1976) Oxygen inhibition of nitroreductase: Electron transfer from nitro radicals–anions to oxygen. *Biochem. Biophys. Res. Commun.*, **69**, 942.